# Hyperspectral Reflectance Response of Wild Rocket (*Diplotaxis tenuifolia*) Baby-Leaf to Bio-Based Disease Resistance Inducers Using a Linear Mixed Effect Model

**DOI:** 10.3390/plants10122575

**Published:** 2021-11-25

**Authors:** Catello Pane, Angelica Galieni, Carmela Riefolo, Nicola Nicastro, Annamaria Castrignanò

**Affiliations:** 1 Council for Agricultural Research and Economics (CREA), Research Centre for Vegetable and Ornamental Crops, Via Cavalleggeri 25, 84098 Pontecagnano Faiano, Italy; nicola.nicastro@crea.gov.it; 2 Council for Agricultural Research and Economics (CREA), Research Centre for Vegetable and Ornamental Crops, Via Salaria 1, 63030 Monsampolo del Tronto, Italy; angelica.galieni@crea.gov.it; 3 Council for Agricultural Research and Economics (CREA), Research Centre for Agriculture and Environment, Via Celso Ulpiani 5, 70125 Bari, Italy; carmela.riefolo@crea.gov.it; 4 Department of Engineering and Geology (InGeo), “Gabriele D’Annunzio” University of Chieti-Pescara, Via dei Vestini 31, 66013 Chieti, Italy; acastrignano53@gmail.com

**Keywords:** proximal sensing, *Trichoderma*, laminarin, yeast cell wall extract, mixed models

## Abstract

Baby leaf wild rocket cropping systems feeding the high convenience salad chain are prone to a set of disease agents that require management measures compatible with the sustainability-own features of the ready-to-eat food segment. In this light, bio-based disease resistance inducers able to elicit the plant’s defense mechanism(s) against a wide-spectrum of pathogens are proposed as safe and effective remedies as alternatives to synthetic fungicides, to be, however, implemented under practical field applications. Hyperspectral-based proximal sensing was applied here to detect plant reflectance response to treatment of wild rocket beds with *Trichoderma atroviride* strain TA35, laminarin-based Vacciplant^®^, and *Saccharomyces cerevisiae* strain LAS117 cell wall extract-based Romeo^®^, compared to a local standard approach including synthetic fungicides (i.e., cyprodinil, fludioxonil, mandipropamid, and metalaxyl-m) and a not-treated control. Variability of the spectral information acquired in VIS–NIR–SWIR regions per treatment was explained by three principal components associated with foliar absorption of water, structural characteristics of the vegetation, and the ecophysiological plant status. Therefore, the following model-based statistical approach returned the interpretation of the inducers’ performances at field scale consistent with their putative biological effects. The study stated that compost and laminarin-based treatments were the highest crop impacting ones, resulting in enhanced water intake and in stress-related pigment adjustment, respectively. Whereas plants under the conventional chemical management proved to be in better vigor and health status than the untreated control.

## 1. Introduction

Wild rocket (*Diplotaxis tenuifolia* (L.) DC) is a cruciferous perennial herb, spontaneous in the Mediterranean Basin. In the last 25 years, Italy has become one of the major European producers of wild rocket using the species in intensified cultivation systems devoted to harvesting fresh-cut baby-leaf for the high convenience salad chain. It is sown with precision seed drills on 1.8–2.2 m width beds under polytunnels, fertigated with sprinklers and mechanically cut at complete foliar development. The harvested product quickly enters the cold chain, is minimally processed by washing, wiping, and bagging, and distributed through retail nets across several countries as ready-to-eat preparations. Packaged wild rocket meets the consumer preferences for the characteristic pungent–aromatic flavor associated with glucosinolates [1] and a few other nutraceutical properties (i.e., vitamins, antioxidants, fibers, and low calories) [2].

This makes the market very sensitive to the sustainability levels of the production process from field to shelf, conceived with a considerable reduction in the applied synthetic fungicides [3,4]. Nevertheless, this vegetable crop as well as all the other baby leaf species is susceptible to a plethora of both specific and non-specific pathogens that significantly reduce yields and impair their quality. As a consequence, the deployment of non-traditional effective control measures that include biological strategies is necessary [5].

Biologically-based disease resistance inducers may be biomimetic compounds or substances sourced from plants or microbes, or non-pathogenic microorganisms capable of eliciting the plant’s own defense mechanism(s) through microbe/pathogen-associated molecular patterns and/or the recognition of host-derived damage-associated molecular patterns to enhance their innate defense response against upcoming broad-spectrum diseases to varying degrees [6,7,8]. Therefore, innovative resistance activators can then be used as biopesticides in plant protection protocols as a safer alternative to synthetic chemicals to reduce the environmental disease management footprint and stimulate plant performances [9]. However, they still need to improve their efficacy under field conditions [10].

Non-invasive technologies may be helpful to optimize the field applications of these plant-targeted protectants [11]. Plants react to exogenous application of plant resistance inducers by possibly activating many metabolic pathways involved in biochemical and mechanical defense responses including shifts of cytosolic ion content, oxidative burst, synthesis of enzymes, proteins, and other secondary metabolites related to the defense, in addition to the activation of resistance-related hormones [12]. Changes in leaf composition and plant health may be detected in the reflected electromagnetic radiation once it is captured by optoelectronic sensors such as hyperspectral ones.

In optics, reflectance measures the ability of a given surface or material to send back part of the incident light on it. In particular, a hyperspectral sensor can record the part of the electromagnetic radiation of a natural (sun) or artificial light source that is reflected by a leaf at a very fine spectral resolution in the range of wavelengths between 350–2500 nm. The spectrum is divided into three regions called, in sequence: Visible (VIS), between 350 and 750 nm; Near Infrared (NIR) until 1400 nm; and the Short-Wave Infrared (SWIR) region until 2500 nm. Each region has been associated with many parameters describing the plant status [13]. Hyperspectral data analysis represents a very effective and sustainable tool for evaluating changes induced in the plant by abiotic and biotic stresses. Recently, hyperspectral data have been adopted on a large scale in the detection of biotic stresses on plants as in the case of the sudden spread of Xylella infection on entire olive cultivations in the Mediterranean area [14,15,16]. Some vegetation indices based on reflectance data in the VIS range have been used to assess changes occurring in the plant health status through the related effect on pigments such as carotenoids, anthocyanins, and chlorophyll. However, these variations might be due to either specific or non-specific infections [17,18,19,20,21], hence further laboratory phytopathogenic analysis on the leaves remains necessary.

The transition from low reflectance values in the red to high values in the infrared spectral range is very rapid: this portion of the spectrum, called Red Edge, is more indicative of the chlorophyll content than that of water [22,23,24,25]. Moreover, it is influenced by the cell structures of leaves that poorly absorb in the NIR because of the multiple scattering of radiation by the mesophyll. As for SWIR, the overlap between information on water content and organic compounds makes data interpretation more difficult. Statistical processing [26], mathematical regressions [16], and radiative transfer models [27,28,29] have been used for this purpose.

This leads to hyperspectral data being used as indicators of possible stress in the plant, although a direct relationship between the alteration observed in the spectra and its cause has not been discovered yet.

The analysis and interpretation of spectral data are further complicated by the way in which agronomic trials are generally conducted. The purpose of the agronomic experiments is to test whether the compared treatments (in this case the use of different plant resistance inducers) have any effect on the supposed response variable. If one treatment is taken as a control (e.g., zero treatment), the experiment will consist of testing whether every other treatment has an effect compared to the control treatment. The biggest challenge in an agronomic trial is to be able to separate the intrinsic variation of the response variable from that induced by the experimental treatments. In traditional agronomic trials, this is achieved by replicating each treatment according to a well-defined experimental design. Traditional statistical methods based on the design then allow for the determination of the probability that any measured difference between treatments is due to chance (null hypothesis). 

Many times, when the experiment is conducted in a confined space such as a greenhouse, or on-farm, for purely practical reasons, there is a tendency to follow a more systematic pattern, with one treatment, for example, assigned to a particular part of the field. In addition, there may be too few plots per treatment (repetitions) to assess the underlying variability, and furthermore, such variability may be correlated [30].

These experiments very often fail to meet the fundamental assumptions required by classical statistical methods. It is therefore necessary to use more complex statistical methods [31,32] that are based on a model-based statistical approach [33]. This consists in describing both the variation and the correlations between the observations of the response variable using a statistical model. In this regard, the theory of linear mixed effects models (LMM) [34,35,36] allows for the total variance to be broken down into that which is attributable to fixed effects, corresponding to the treatments, and that which is attributable to random effects. The latter are linked to the intrinsic spatial variability of the agronomic system, which cannot be described by fixed effects and can be estimated by the covariance/correlation function of residuals.

Therefore, with a view to increasing sustainability in greenhouse cultivations of wild rocket, the use of naturally derived products can be proposed, but their actual interaction with the plants must be continuously monitored. For this purpose, among micro- and macroorganisms, certain active components described as plant defense promoters can be found and formulated in some successful experimental and commercial products to induce resistance in the plants. These may differ in the level of purity of the active ingredients, ranging from microorganisms to a single molecule, in the hormonal signaling pathways involved in the elicited plant reactions (i.e., oxidative burst, cell-wall fortification, etc.) and in the ranges of efficacy. Furthermore, proper organic soil management with compost amendments that promote plant growth and development by improving soil chemical, physical, and microbiological quality and fertility can synergize with inputs to the aerial part, leading to greater beneficial effects. 

The specific objective of the work is to characterize the hyperspectral response of greenhouse-grown wild rocket to the application of three immunity-stimulating active ingredients (resistance inducers), split over plots amended and non-amended with green compost, using the mixed effect model theory to account for the actual experimental conditions.

## 2. Results

### 2.1. Principal Component Analysis (PCA)

Four principal components (PCs) with eigenvalues greater than 1 were extracted. Among these, however only three PCs explaining a cumulative variance proportion greater than 97% were retained (Table 1). 

The most relevant bands for PC1 fell within the ranges 1405–1545 nm and 1855–2500 nm with a small plateau at 1435–1475 nm, a peak at 1885 nm, and two other plateaus at 2055–2105 and 2345–2385 nm. Since water peaks fall in SWIR regions (wavelengths centered at about 1450, 1940, and 2200 nm), PC1 appears to be related to foliar absorption of water [29,37]. However, these absorbance bands may also be related to the characteristics of chemical components such as cellulose, starch, and proteins. In particular, the peak at 1885 nm could be attributable to a stretch absorption mechanism of chemical groups such as OH and CO in cellulose and hemicellulose, and the plateau at 2345–2385 seems to be linked to a deformation mechanism of the CH bond [27]. The range 2055–2105 nm was highly correlated with N content [38], while the range 1435–1475 nm overlapped with the main absorbance peak of water at 1450.

PC1 could therefore be used as an indicator of the leaf water status as well as the content of cellulose, starch, and proteins, whose variations might be caused by mechanisms of action of the inducers through the biochemical pathways they influence (i.e., the closure of stomata) [39,40,41,42]. It is worth noting that since the data refer to reflectance, the relationship between PC1 and water, cellulose, starch, and protein contents is inverse.

Regarding PC2, the main loadings fell within the range 715–1375 nm. More specifically, the subrange up to 935 nm was more related to the structural characteristics (LAI) of the vegetation [22]. The secondary water absorption peak at 970 nm, often used to compute vegetation indices of water content [43], fell into the next subrange, as did the other peculiar water absorbance peak at 1200 nm [44]. PC2 can therefore be interpreted primarily as an indicator of the structural characteristics of the leaf cells and only secondarily as an indicator of the water content. Unlike PC1, the relationship between PC2 and LAI is direct as it is well-known that better leaf vigor produces higher reflectance in the NIR [22].

**Figure 1:**
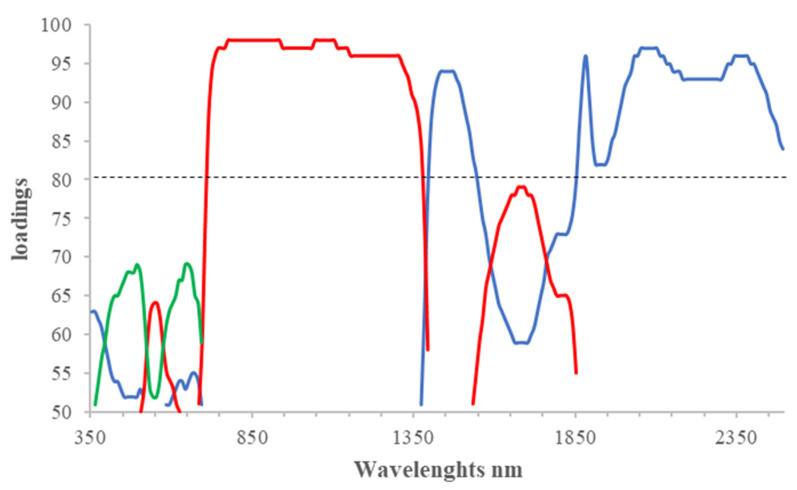
Graph of the loadings of the first PC1 (blue line), the second PC2 (red line), and the third PC3 (green line) >80. The dashed line represents the chosen threshold for loading values.

PC3 was considered, despite the loadings not exceeding the established limit value of 80, because of the additional information it can provide regarding the spectral response of wild rocket to the action of inducers. The path of its loadings (Figure 1) showed two peaks between 365–695 nm in VIS. The first peak had a maximum at 495 nm, a value related to the carotenoid content as an indicator of plant stress [45,46], while the second peak at 650 nm was related to chlorophyll content, as green plants have a main absorption peak in the red. In fact, the two leaf contents are closely related and their Car/Chla ratio can be used as an indicator of the ecophysiological state of leaves and plants [47]: a high ratio denotes severe stress.

Therefore PC3, despite the small portion of variance explained, can be taken as an indicator of plant health as high reflectivity in this VIS range might be associated with a low carotenoid content, intended as an indicator of stress, and efficient chlorophyll function.

### 2.2. Linear Mixed Model (LMM)

Before any further processing, the first three principal components, standardized to mean 0, were tested against the assumption of normal distribution. From the examination of Table 2, only PC2 showed significant deviations from normality, as can be verified by the results of the basic statistics and normality tests. It was therefore necessary to perform the relative rank transformation only for PC2, since the pronounced skewness of its distribution might affect the linearity of the mixed model to be estimated. From this point onward, all statistical processing is understood to be carried out on the rank-transformed PC2 values (rPC2) and the raw values of PC1 and PC3.

The results of the Levene’s test applied to the three variables are shown in Table 3. Assuming a reference probability level of 0.05, the fixed effect COMPOST is non-homogeneous only for PC1, while the effect TREATMENT is non-homogeneous for PC1 and PC3.

Both Moran’s I test and Geary’s c test were significant at *p* < 0.05 for all three variables (Table 4). However, these are overall tests: more information on the type and intensity of the spatial association can be obtained from residual-fitted variogram models.

Regarding the residual variogram models, a spherical model with nugget was fitted for PC1 and PC3 and a pure nugget effect model for rPC2 (Table 5). Although the parameters of the variogram models were significantly different from zero, it can be seen that the nugget effect for PC1 and PC3 was 87% and 96%, respectively, of the total sill (nugget + partial sill). These results show that although spatial autocorrelation occurred for PC1 and PC3, the structured component (partial sill) was only a small percentage (13% and 4%, respectively) of the total spatial variance because the spatially uncorrelated component (nugget effect) was highly prevalent. This can be explained based on the particular experimental scheme in which an intrinsic variability of the plant–soil system was superimposed on an artificial variability imposed by man through randomization in treatment assignment. However, these variogram models were only used to initialize the iterative process for jointly estimating the random effects parameters and fixed effects coefficients of the mixed effects models to be determined.

Table 6 shows the results of the mixed-effects model estimation for PC1, separated between the estimates of the stochastic effect parameters (Table 6a) and the statistical significance of the fixed effects (Table 6b). Regarding the variogram of the residuals, two separate models were applied for the two levels of the COMPOST effect due to the heteroscedasticity of variance. For the NO level. a pure nugget effect was fitted, revealing a lack of spatial structure. For the YES level, it was possible to fit a spherical model, which, when compared with the previous least squares model (Table 5), had a shorter interval but a higher partial sill. In any case, it represents a short-range correlation with a spatially uncorrelated component more than twice as large as the structured one. 

Unlike for the TREATMENT effect, it was not possible to fit five separate variogram models due to insufficient degrees of freedom because of the small number of observations/replications.

Regarding the fixed effects, both main effects and their interaction were highly significant.

Only the most significant differences between the various levels of the two effects and their interaction at *p* < 0.05 are shown in Table 7.

For the COMPOST effect, the mean of the NO level for PC1 was larger than the mean of the YES level. Recalling the meaning attributed to PC1, it can be stated that plants treated with compost might have a higher water content. 

For the effect of TREATMENT, CTR mean was significantly lower than that of the CER, TRI, and CHE levels and therefore might be associated with higher water content.

For rPC2, it was possible to fit a spherical model with nugget for the random effect (Table 6), in contrast to the least squares model (Table 5), which was the pure nugget effect. However, once again, this was short-range variability (<3 m) and with a spatially structured component that was only 14% of the spatially uncorrelated.

Regarding the fixed effects, only TREATMENT was significant, and in Table 7, only the differences between the TREATMENT levels that were significant are shown. Based on the interpretation of rPC2, which correlated positively with LAI, we can state that CHE treatment showed better leaf vigor than all remaining levels.

For PC3, there was sufficient agreement between the least squares estimates (Table 5) of the random effect and the RML estimates (Table 6a). However, in the latter case, there was a slight increase in the spatially structured component, which remained a small part (11%) of the spatially uncorrelated component. Both fixed effects and their interaction were highly significant (Table 6b).

Based on the interpretation of PC3, plants treated with compost appeared to be healthier than untreated plants and the same was true for plants under CHE treatment compared to those in the other levels (Table 7). These results are consistent with those obtained with rank transformed PC2.

In summary, the above analysis showed that the compost-treated plants had a higher water content than untreated plants. Similarly, the plants in level CTR of TREATMENT appeared wetter than those in the remaining levels.

In contrast, the plants in CHE appeared to be more luxuriant and healthier than those in the other levels. Statistical analysis showed that the apparently winning combination (YES-CHE) differed significantly for both PC1 and PC3 from the combinations YES-CTR and NO-CTR, resulting in lower water content but lower stress level (Table 7).

The results of the traditional analysis of variance, assuming that the residuals were spatially uncorrelated, are also shown for comparison in Table 8. Indeed, there were no significant differences and this was quite consistent with what was expected, given the small proportion of the structured component of the variance compared to the spatially uncorrelated component. However, the values of the F statistic tended to be higher than those of the mixed effects models, underlining the risk of committing type I errors if the spatial correlation is neglected [48].

## 3. Discussion

The statistical results obtained are consistent with a biological interpretation, which reinforces the idea that the spectral response of the plant can be used as an effective and reliable indicator of its health. PC1 summarizes information about N content and other biochemical compounds, which to date, has come from several studies regarding the SWIR region on potato and other mapped vegetation [49,50]. As far as PC2 is concerned, the main loadings fell in the ranges more related to the LAI as confirmed by recent studies on rice and maize in both proximal and remote sensing [51,52], and to the water absorption peaks that are used to calculate new vegetation indices associated with water content in different plant species [53]. Finally, PC3 can explain the ratio Car/Chla as an indicator of plant stress [54].

It is generally recognized that the incorporation of compost into the soil increases the water available to plants [55], delaying the possible wilting associated with drought [56] and thus protecting and/or enhancing photosynthetic activity [57]. On the other hand, the lack of statistical significance of the COMPOST effect for rPC2, which was associated with plant vigor, can be explained on the basis of the reduced nutrient supply capacity shown by green composts in the presence of a large fraction of non-labile carbon, whose degradation implies the net immobilization of N [58]. In addition, compost in combination with LAM and TRI had a positive effect on the water content (PC1) and in combination with LAM and CHE on the general health of the plant (PC3). In contrast, compost in combination with CER did not produce any positive effects in terms of either water content or LAI. 

However, the resistance inducers used in this study, on the basis of their specific characteristics, might be implicated in the physiological processes underlying the interpretation of the PCs. Antagonistic fungi belonging to the genus *Trichoderma* have been reported to induce a resistance response into plants through multiple hormonal signaling pathways that modulate jasmonic acid, ethylene, and salicylic acid levels toward a wide-spectrum of pathogens [59]. Their biocontrol efficacy might result in the modulation of plant growth and yield improvement [60]. Kumar and Kumar [61] reported that root colonization of *Trichoderma* sp. can induce the production of stress enzymes such as peroxidase and glutathione reductase, which may be responsible for decreasing disease incidence in *Brassica juncea*. In a different way in cabbage, *Trichoderma* treatments increased the transcript levels of genes related to photosynthesis and sucrose transport, PR proteins, chitinases, and oxidases [62]. Yeast cell-wall extract, which carries polysaccharidic and peptidic polymers and oligomers of highly variable molecular mass, acts as MAMPs in inducing defense-related events through SA signaling [40,41,42,63]. However, there is no evidence in the literature that it has an impact on the reflectance of plants. On the other hand, concerning laminarin, which is a water-soluble glucan storage polysaccharide extracted from brown algae (i.e., *Laminaria digitata* Hudson, Lamouroux), it has been shown that it can elicit defense reactions in several plant species [64] via salicylic acid and reactive oxygen species pathways [65]. This is most likely due to the association with bound β-1,3–1,6 glucosyl residues [39]. It is also worth pointing out that in this study, the LAM effect on TRI was significantly higher in all pairwise COMPOST × TREATMENT interactions relative to PC3 associated with indicating stress occurrence. Consistently, laminarin has been reported as an unconventional elicitor of plant secondary metabolites [66]. In *Arabidopsis*, this molecule increased chloroplast stability by activating the antioxidant system under stress conditions [67]. Consequently, with regard to PC3, the current hyperspectral study indicated that LAM treatment associated with the compost effect is linked to the improved plant health status.

## 4. Materials and Methods

### 4.1. Experimental Design

The trial was conducted under a multi-tunnel greenhouse located at the CREA-Research Center for Vegetable and Ornamental Crops (Pontecagnano Faiano, Italy, 40°38′54.0″ N 14°53′21.4″ E). Wild rocket cv Yeti (Maraldi Sementi, Italy), suitable for the winter–spring cropping cycle, was precision sown on 19 November 2019 (2500 seeds m^−2^) and grown on 1.3 m wide beds under two polyethylene tunnels (20 × 7.2 m) with four beds each equally spaced by 0.5 m. 

**Figure 2:**
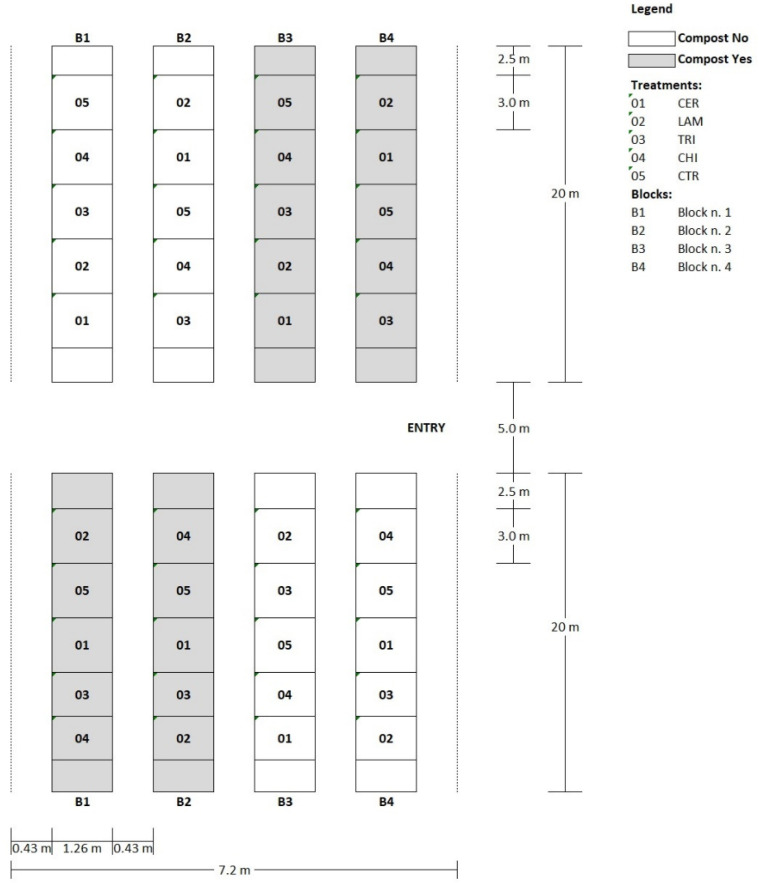
Experimental design of the two-way split-plot field trial carried out under a multi-tunnel greenhouse located at the CREA-Research Center for Vegetable and Ornamental Crops.

**Figure 3:**
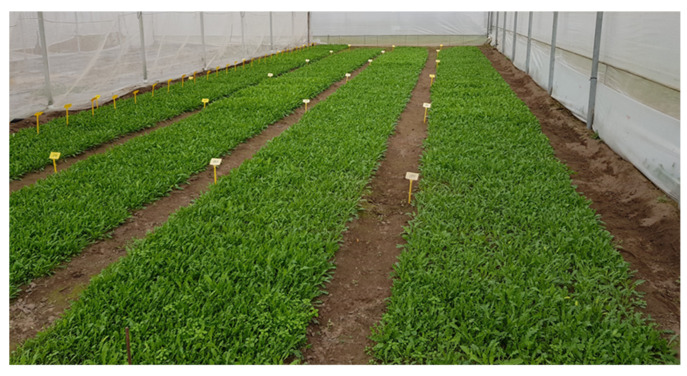
Experimental field trial consisting of a baby-leaf wild rocket cultivation carried out under greenhouse located at the CREA-Research Center for Vegetable and Ornamental Crops.

The experimental design was a two-way split-plot with the external effect (COMPOST) represented by the application or not of green compost as an amendant (two levels: Yes/No). The internal effect (TREATMENT) was represented by the application of inducers including five treatments: (1) two applications (at seeding and pre-emergence) of *Trichoderma atroviride* strain TA35 from CREA Collection [68] at a dose of 1000 L ha^−1^ (10^5^ cells mL^−1^) (TRI); (2) four weekly spray applications of commercial laminarin-based product (Vacciplant^®^, Arysta Lifescience s.a.s., France) at dose of 1 L ha^−1^ (LAM); (3) four weekly spray applications of *Saccharomyces cerevisiae* strain LAS117 cell wall extract-based commercial product (Romeo^®^, Agrauxine, France) at a dose of 750 g ha^−1^ (CER); (4) local standard approach including one spray with Cyprodinil, Fludioxonil, Mandipropamide, and Metalaxil-M (CHE); and (5) no treatment (CTR) used as reference control. Main plots were not-amended or amended with green compost (10 t ha^−1^ dry matter) (COMPOST). The experimental design consisted of four blocks (each one including a single bed but split into two tunnels North/South); each block was split longitudinally into two halves randomly assigned to one level of COMPOST; each half of the block was randomly split into the five levels of the internal effect. The trial then consisted of 40 experimental units (plots) with a size of 3.0 × 1.3 m (Figure 2 and Figure 3).

### 4.2. Hyperspectral Reflectance Measurements

Leaf spectral measurements were performed in the spectral range 350–2500 nm, using a portable non-imaging spectroradiometer (FieldSpec^®^ 4 Hi-Res, ASD Inc., Boulder, CO, USA) through a fiber-optic contact probe (ASD Plant Probe; ASD Inc., USA) with a spectral resolution of 3 nm in VIS-NIR and 8 nm in SWIR, a 10 mm field of view, and an integrated halogen reflector lamp. The instrument was warmed up for 90 min before measurements to increase the quality and homogeneity of acquired data. Calibration was obtained with the pre-calibrated white 99% spectral reference panel. Each sample scan represents a mean of 10 reflectance spectra. 

Within each plot, 12 spots spaced 50 cm apart were selected based on the orientation of the leaves in sunlight, with preference given to the top and most exposed leaves; the reflectance measurement for each spot was obtained as an average of three replicates. The reflectance data were then averaged over 10-nm intervals, thus reducing the number of wavelengths from 2151 to 215, smoothing the spectra and keeping the risk of over-fitting low [69]. Pre-processing methods were applied to reduce the impact of multiplicative and additive effects of possible backscattering within the instrument [70]. The pre-processing first involved the splice correction, in order to minimize the inconsistency recorded at the spectral intervals among the three detectors of the spectroradiometer: the VIS-NIR range 350–1000 nm, which is characterized by a sampling interval of 1.4 nm and the two ranges in the NIR-SWIR, 1000–1800 nm and 1800–2500 nm, with a sampling interval of 2 nm. After that, two pretreatments were performed on the reflectance spectra: (1) multiple scattering correction (MSC); and (2) smoothing/denoising with Savitzky–Golay polynomials [71]. Multiple scattering correction (MSC) works mainly when the scattering effect is the dominant source of variability and removes additive and multiplicative components [72]. 

The first order Savitzky–Golay (SG) polynomial algorithm reduces the random noise of the measurements. The algorithm is based on a moving polynomial fit of any order and the filter size consists of (2n + 1) points, where n is the half-width of the smoothing window (w). The polynomial fit interpolates the points between the 2n’s. A window size (w) of 11 (w = 2n + 1) and the second polynomial order were applied here [73].

Splice correction was achieved using ViewSpecPro software (Analytical Spectral Devices Inc., Boulder, CO, USA). The other preprocessing methods were performed using ParLeS software [74].

### 4.3. Principal Component Analysis

Principal component analysis is a widely used dimensionality reduction technique that allows for the extraction of principal components (PCs), expressed as a linear combination of variables [75,76]. The PCs found in this way do not represent directly observable variables and must therefore be interpreted from a scientific-rational perspective. Mathematically, PCA works on the correlation matrix and extracts a few PCs equal to the number of measured variables, but only a part of them is useful.

The number of PCs was fixed by selecting the PCs with eigenvalues greater than or equal to 1 (Kaiser’s criterion) [77]. The eigenvalues refer to the share of variability “explained” by the PC and take on descending values as the first PC moves toward the last one. The result can then be subjected to rotation by various methods. Methods using orthogonal rotations preserve the independence of the PCs. The VARIMAX procedure was used in this study.

The most important parameters to evaluate were:

The amount of variance “explained” by both the set of the retained PCs (cumulative) and by each PC individually;

PC loadings, which describe the strength of the relationship between the PC and the variable being measured.

PCA was performed with the SAS/FACTOR procedure of the statistical software package SAS/STAT (release 9.4 SAS ANALYTICS U software). 

For each PC, the loading values were multiplied by 100. The value of 80 was chosen as a limit above which to select the most relevant wavelengths. 

The raw spectral data were preferred over the pretreated ones because with the same number of PCs, they explained a greater proportion of variance, probably due to the specific measurement modalities of the plant probe that reduced the random error.

### 4.4. Statistical Analysis

The assumption of univariate normality for each PC was checked with three non-parametric tests (Kolmogorov–Smirnov, Cramer-von Mises, and Anderson–Darling) [78].

If the variable showed large departures from normal distribution, to improve the linearization of the mixed effect model, PC was transformed to normal scores (*y_i_*) using Blom’s formula [79]:

$$y_{i}=\Phi^{-1}\frac{\left(r_{i}-3/8\right)}{\left(n+1/4\right)}$$

where Φ^−1^ is the inverse cumulative normal (PROBIT) function; *r_i_* is the rank of the *i*th observation; and *n* is the number of observations that have non-missing values for the ranking variable.

The spatial association of the residuals from the OLS models, obtained assuming independence of the residuals, was verified in two ways:

(1) By calculating the spatial autocorrelation statistics of Moran (1950) [80] and Geary [81] and comparing them to the null hypothesis statistics of completely random spatial model; and

(2) By fitting an authorized mathematical model to the experimental variogram of the OLS residuals, according to the least squares estimation (LSE) technique and testing the statistical significance of its parameters.

### 4.5. Linear Mixed Effect Model (LMM)

A LMM can be written in the form:

**z** = **Mβ** + **ε**

where **z** is a vector of length n corresponding to the measurements of the response variable; and **M** is the fixed effects design matrix of size n × t, where t is the total number of different experimental treatment levels. In the case under study, there are two levels for the COMPOST effect, five for the TREATMENT, and 10 for the interaction effect. **β** is the vector of length t of the coefficients of the fixed effects and ε the vector of length n of the random effects. The product **Mβ** represents the fixed effects. All entries of the matrix **M** (M_ij_) can assume the values 0 or 1. The entries of the first column (M_1j_) are all equal to 1 while the other Mij entries are 1 if observation i corresponds to the treatment level j, otherwise they are 0. Therefore, β_1_ corresponds to the overall mean and the other β_j_ correspond to the adjustment of this mean concerning each treatment level j. The elements of the vector **ε** (e_i_), also known as residuals, are assumed to follow a normal distribution with mean 0 and covariance matrix Cov[e_i_, e_j_]. This matrix may differ from the identity matrix, and the random effects may also be spatially correlated.

Usually, the covariance is assumed to be a function of the distance between the locations x_i_ and x_j_. If by **h** we denote the distance between x_i_ and x_j_, the covariance model takes the general form:

Cov[e_i_, e_j_] = c(**h**) = σ [f(**h**)]

where σ is known as the partial sill and with the symbol f, one of the authorized mathematical functions used to represent a covariance function, is indicated. The models, for which f(**h**) is the same for all pairs of observations equally distant in a given direction, are called stationary models (of the second order if the average, assumed constant, is known). If f(**h**) does not depend on direction, then the covariance structure is said to be isotropic. Many isotropic covariance models exist, however, in this study, we used the spherical model:

c(h) = σ 0 + σ [1 − 1.5(h/ρ) + 0.5(h/ρ)3]  if h< ρ

c(h) = 0  if h≥ ρ

This model has three parameters that must be determined from the data: the nugget variance (σ 0), the partial sill (σ)m and the range ρ. These spatial models are drawn from geostatistics and refer to one of the numerous manuals [82,83,84] for an interpretation of these parameters. It is commonly preferred to use the variogram instead of the covariance function given by the formula:

γ (**h**) = σ 0 + σ − c(**h**)

Furthermore, complexity in determining the covariance model may arise from the heteroscedasticity of the variance relative to the individual PCs tested, as this may result in heterogeneity in the covariance function. The homogeneity of the variance was tested with Levene’s test [85]. 

The method of fitting the spherical model to the values of mean covariance for each class of distance, **h**, is based on an iterative procedure, aimed at maximizing the log likelihood of the residuals using the restricted maximum likelihood method (REML) [86]. Once the spatial covariance function is estimated, the fixed effects coefficients are obtained as generalized least squares estimates [86]. For comparison, traditional models (OLS), which assume residuals are normally distributed with zero mean but not correlated spatially, were also determined.

All statistical analyses were performed using statistical software package SAS/STAT (release 9.4 SAS ANALYTICS U software) and in particular, LMMs were estimated using the procedure MIXEDe.

## 5. Conclusions

This study demonstrated that hyperspectral proximal sensing of the wild rocket crop is suitable for tracking the field performance of canopy treatments with resistance inducers, based on their specific interactive mechanisms with the plant. Compost and laminarin-based treatments had the greatest impact on the crop, resulting in increased water uptake and in stress-related pigment regulation, respectively. Ultimately, the plant under the conventional chemical management proved to be more vigorous and healthier than the untreated control.

Furthermore, in order to produce reliable results in agronomic trials that are actually useful to farmers, knowledge of the spatial variability of the response variable can no longer be ignored. Otherwise, there is a risk of assigning to the experimental factor an effect that is instead attributable to the intrinsic variation of the crop–soil system. It is hoped that the theory of mixed effects models will become increasingly familiar in the agronomic community.

## Figures and Tables

**Table 1:** Basic statistics of the PCs.

Variables	Mean	Median	Std Deviation	Skewness	Kurtosis
PC1	−0.0455	−0.0594	0.697	0.358	0.230
PC2	−0.0641	0.0823	0.769	−1.183	2.185
PC3	0.011	0.0003	0.805	0.297	0.671

**Table 2:** Normality tests of the PCs.

Test	Statistic	PC1	PC2	PC3	*p*-Value	PC1	PC2	PC3
Kolmogorov-Smirnov	D	0.030	0.100	0.032	*p* > D	>0.1500	<0.0100	>0.1500
Cramer-von Mises	W-Qu	0.095	1.557	0.048	*p* > W-Qu	0.1352	<0.0050	>0.2500
Anderson-Darling	A-Qu	0.701	8.887	0.393	*p* > A-Qu	0.0707	<0.0050	>0.2500

**Table 3:** Levene’s test of variance homogeneity for PC1, Gaussian transformed PC2 (rPC2) and PC3.

Effects	DF	PC1		rPC2		PC3	
		F Value	*p* > F	F Value	*p* > F	F Value	*p* > F
Compost	1	5.14	0.0238	0.47	0.4919	0.73	0.3931
Treatment	4	4.07	0.0030	1.08	0.3648	1.14	0.3352

**Table 4:** Tests of spatial autocorrelation of the three PCs.

Variable	Coefficient	Observed	Expected	Std Dev	Z	*p* > |Z|
PC1	Moran’s I	0.094	−0.002	0.025	3.85	0.0001
	Geary’s c	0.902	1.00	0.026	−3.73	0.0002
rPC2	Moran’s I	0.065	−0.002	0.025	2.69	0.0071
	Geary’s c	0.932	1.00	0.026	−2.62	0.0089
PC3	Moran’s I	0.050	−0.002	0.025	2.08	0.0373
	Geary’s c	0.941	1.00	0.026	−2.23	0.0256

**Table 5:** LSE estimates of the variogram parameters for PC1, rPC2, and PC3.

	PC1			rPC2			PC3		
Parameter	Value	t Value	*p* > |t|	Value	t Value	*p* > |t|	Value	t Value	*p* > |t|
Nugget	0.376	54.78	<0.0001	0.917	250.62	<0.0001	0.556	90.35	<0.0001
Partial sill	0.053	7.12	<0.0001	-	-	-	0.022	2.83	0.0115
Range (m)	6.41	11.98	<0.0001	-		-	11.19	6.63	<0.0001

**Table 6:** Results of the mixed effect model estimation for the PCs.

**a. Random Effect**						
**Variable**	**Variogram Model Parameters**	**Effect**	**RML Estimate**	**Standard Error**	**z Value**	***p* > Z**
PC1	Nugget	Compost NO	0.259	0.059	4.42	<0.0001
	Partial sill	Compost YES	0.100	0.042	2.40	0.0081
	Range (m)	Compost YES	2.77	0.840	3.30	0.0005
	Nugget	Compost YES	0.256	0.034	7.62	<0.0001
rPC2	Partial sill		0.114	0.062	1.84	0.0332
	Range (m)		7.25	3.03	2.39	0.0084
	Nugget		0.898	0.064	14.09	<0.0001
PC3	Partial sill		0.062	0.042	1.48	0.0689
	Range (m)		11.18	6.68	1.67	0.0470
	Nugget		0.557	0.038	14.46	<0.0001
**b. Fixed Effect**						
	**PC1**		**rPC2**		**PC3**	
**Effect**	**F Value**	***p* > F**	**F Value**	***p* > F**	**F Value**	***p* > F**
Compost	13.40	0.0015	0.04	0.8507	6.33	0.0185
Treatment	3.17	0.0155	3.39	0.0106	4.92	0.0008
Compost × Treatment	4.42	0.0021	0.99	0.4145	3.87	0.0050

**Table 7:** The most relevant significative LSE differences between the levels of each effect and the ones of interaction.

Variables	Effect	Compost	Treatment	Compost	Treatment	Estimates	Standard Error	t Value	Pr > |t|
PC1	Compost	NO		YES		0.305	0.083	3.66	0.0015
	Treatment		CER		CTR	0.221	0.111	1.99	0.0486
	Treatment		TRI		CTR	0.298	0.110	2.71	0.0076
	Treatment		CHE		CTR	0.376	0.111	3.37	0.0010
	Compost × Treatment	NO	LAM	YES	TRI	0.4803	0.1630	2.95	0.0041
	Compost × Treatment	YES	LAM	NO	TRI	−0.6451	0.1630	−3.96	0.0002
	Compost × Treatment	NO	CHE	YES	CTR	0.396	0.163	2.43	0.0170
	Compost × Treatment	YES	CHE	NO	CTR	0.356	0.163	2.18	0.0318
	Compost × Treatment	YES	CHE	YES	CTR	0.548	0.168	3.27	0.0019
rPC2	Treatment		CER		CHE	−0.604	0.166	−3.64	0.0003
	Treatment		LAM		CHE	−0.357	0.160	−2.24	0.0262
	Treatment		TRI		CHE	−0.332	0.157	−2.12	0.0356
	Treatment		CHE		CTR	0.346	0.160	2.17	0.0321
PC3	Compost	NO		YES		−0.259	0.103	−2.52	0.0185
	Treatment		CER		CHE	−0.367	0.126	−2.92	0.0040
	Treatment		LAM		CHE	−0.351	0.121	−2.90	0.0041
	Treatment		TRI		CHE	−0.500	0.119	−4.18	<0.0001
	Treatment		TRI		CTR	0.264	0.122	2.17	0.0313
	Compost × Treatment	YES	LAM	NO	TRI	0.5532	0.1842	3.00	0.0034
	Compost × Treatment	YES	LAM	YES	TRI	0.5248	0.1760	2.98	0.0033
	Compost × Treatment	YES	CHE	NO	CTR	0.365	0.182	2.01	0.0478
	Compost × Treatment	YES	CHE	YES	CTR	0.423	0.169	2.51	0.0131

**Table 8:** Results of the mixed effect model estimation for the PCs.

	PC1		rPC2		PC3	
Effect	F Value	*p* > F	F Value	*p* > F	F Value	*p* > F
Compost	25.17	<0.0001	0.53	0.4686	18.64	<0.0001
Treatment	4.91	0.0007	2.41	0.0484	5.18	0.0004
Compost × Treatment	6.34	<0.0001	1.64	0.1640	4.13	0.0027

## Data Availability

The data presented in this study are available in the article.
